# Low prevalence of *mcr-1* in *Escherichia coli* from food-producing animals and food products in China

**DOI:** 10.1186/s12917-024-03891-6

**Published:** 2024-02-01

**Authors:** Cai-Yue Mei, Yue Jiang, Qin-Chun Ma, Meng-Jun Lu, Han Wu, Zhen-Yu Wang, Xinan Jiao, Jing Wang

**Affiliations:** 1 Jiangsu Key Laboratory of Zoonosis/Jiangsu Co-Innovation Center for Prevention and Control of Important Animal Infectious Diseases and Zoonoses, Yangzhou University, Yangzhou, 225009 China; 2 Key Laboratory of Prevention and Control of Biological Hazard Factors (Animal Origin) for Agrifood Safety and Quality, Ministry of Agriculture of China, Yangzhou University, No. 48 Wenhui East Road, Yangzhou, Jiangsu 225009 China

**Keywords:** Colistin, *Escherichia coli*, *mcr-1*, Plasmids

## Abstract

**Background:**

*mcr-1-*positive *Escherichia coli* has emerged as a significant threat to human health, veterinary health, and food safety in recent years. After the prohibition of colistin as a feed additive in animal husbandry in China, a noticeable reduction in both colistin resistance and the prevalence of *mcr-1* was observed in *E. coli* from animals and humans.

**Objectives:**

To assess the prevalence of the colistin resistance gene *mcr-1* and characterize its genetic context in *E. coli* strains derived from fecal and meat samples from food-producing animals in China.

**Methods:**

A total of 1,353 fecal samples and 836 food samples were collected between 2019 and 2020 in China. *E. coli* isolates were identified using matrix-assisted laser desorption/ionization time-of-flight mass spectrometry and their susceptibility to colistin were determined using the broth microdilution method. The colistin-resistant *E. coli* isolates were screened for the presence of *mcr* by PCR analysis and sequencing. The minimal inhibitory concentrations (MICs) of 15 antimicrobial agents against the *mcr-1*-positive strains were further tested using the agar dilution method, conjugation assays were performed, and whole genome sequencing was performed using Illumina HiSeq.

**Results:**

In total, 1,403 *E. coli* strains were isolated. Thirteen isolates from chicken meat (*n* = 7), chickens (*n* = 3), and pigs (*n* = 3) were resistant to colistin with MIC values of 4 to 16 mg/L, and carried *mcr-1*. All *mcr-1*-positive strains, except for isolate AH20PE105, contained multiple resistance genes and exhibited multidrug-resistant phenotypes. They belonged to 10 sequence types (STs), including a novel ST (ST14521). *mcr-1* was located on IncI2 (*n* = 9), IncX4 (*n* = 2), and IncHI2 (*n* = 2) plasmids, which were highly similar to other *mcr-1*-carrying plasmids sharing the same incompatibility type. Seven *mcr-1*-carrying plasmids could be successfully conjugally transferred to *E. coli* C600.

**Conclusions:**

While the low prevalence of *mcr-1* (0.93%) identified in this study may not immediately seem alarming, the very emergence of this gene merits attention given its implications for colistin resistance and public health. Hence, ongoing surveillance of *mcr-1* in *E. coli* remains crucial.

**Supplementary Information:**

The online version contains supplementary material available at 10.1186/s12917-024-03891-6.

## Introduction

The emergence and rapid growth of antibiotic-resistant bacteria pose major challenges to human health, veterinary health, and food safety on a global scale because of the improper use of antibiotics in clinical application and livestock farming [1]. A lack of novel antibacterial agents to combat multidrug-resistant (MDR) Gram-negative bacteria has led to the reuse of polymyxins, particularly colistin, in treatment programs [1]. However, resistance to colistin has been on the rise following the emergence and widespread distribution of the plasmid-borne colistin resistance gene *mcr-1* [1–4]. To date, 10 *mcr* variants and many subvariants have been globally identified in diverse bacterial species, particularly *Escherichia coli*, *Klebsiella pneumoniae*, and *Salmonella* species, from various sources [1, 5]. Among the 10 *mcr* variants, *mcr-1* stands out as the predominant type [1].

*E. coli* are Gram-negative bacteria. Commensal variants of *E. coli* are harmless, whereas pathogenic variants can trigger intestinal or parenteral infections in humans and many animal hosts [6]. Statistically, 48 per 100,000 individuals in high-income countries are diagnosed with *E. coli* bacteremia infections every year [6]. A major concern is that MDR *E. coli* may cause treatment failures because they are a potential reservoir of drug resistance genes, particularly *mcr-1*, which confers resistance to colistin [7]. *E. coli* isolates carrying *mcr-1* prevail significantly among food-producing animals and exhibit widespread distribution across various sources, including humans, wildlife, companion animals, food products, and the environment [1–4, 8]. The rapid dissemination can be attributed to the use of colistin in veterinary medicine or as a growth promoter, along with the capability of *mcr-1* for horizontal transfer [1, 3, 8]. The insertion sequence IS*Apl1*, transposon Tn*6330* (IS*Apl1*–*mcr-1*–IS*Apl1*), and many plasmids (such as IncX4, IncI2, and IncHI2) have been reported to be involved in *mcr-1* transmission among various sources [1, 3, 4].

Following the prohibition of adding colistin to animal feed for growth promotion on April 30, 2017, significant effects have been observed in China, notably in reducing both colistin resistance and the prevalence of *mcr-1*-positive *E. coli* [9]. To further assess the prevalence of *mcr-1*, we examined the dissemination of *mcr-1* in *E. coli* from food-producing animals and retail raw meats and analyzed the genetic environment of *mcr-1* in this study.

## Materials and methods

### **Detection of* mcr* and antimicrobial susceptibility testing**

From June 2019 to November 2020, a total of 1,353 fecal samples were collected from food-producing animals including pigs (*n* = 212), chickens (*n* = 358), cattle (*n* = 752), and pigeons (*n* = 31) from farms located in Anhui, Henan, Liaoning, Jiangsu, Guangdong, Shandong, and Xinjiang in China and 836 food samples including pork (*n* = 377), chicken meat (*n* = 341), and beef (*n* = 118), were collected from a slaughterhouse, farmers’ markets, and supermarkets in the aforementioned provinces and Shanghai (Supplementary Table S1). The isolation of *E. coli* using previously described methods with minor modifications [10, 11]. The samples were cultured for 18–24 h in buffered peptone water (BPW) broth at 37 °C. The positive growth was further streaked on a MacConkey agar plate and incubated at 37 °C for 24 h. One pink colony from each plate was inoculated onto an eosin methylene blue (EMB) agar plate for 24 h at 37 °C. A colony with metallic sheen color (presumptive *E. coli*) was inoculated onto another EMB agar plate for purification. One *E. coli* isolate was randomly chosen from each plate and identified using matrix-assisted laser desorption/ionization time-of-flight mass spectrometry (Bruker Daltonik GmbH, Bremen, Germany).

The susceptibility of all *E. coli* isolates to colistin was assessed using the broth microdilution method according to the International Standards ISO 20776-1 (https://www.iso.org/standard/70464.html). The colistin-resistant *E. coli* isolates were screened for the presence of *mcr* by PCR analysis and sequencing using the primers listed in Supplementary Table S2. The susceptibility of the *mcr*-positive isolates to 15 antimicrobial agents was further assessed using the agar dilution method following the guidelines of the Clinical and Laboratory Standards Institute (CLSI) M07 [12]. The results were interpreted according to the 30th edition of the CLSI M100 [13]. *E. coli* ATCC 25,922 served as the quality control in antimicrobial susceptibility testing.

### Whole genome sequencing and analysis

Genomic DNA was extracted from all *mcr-1*-positive *E. coli* isolates using the TIANamp Bacteria DNA Kit (Tiangen, Beijing, China), according to the manufacturer’s instructions. The Illumina HiSeq platform was used to sequence all *mcr-1*-positive isolates, and SPAdes v.3.8.2 was used to assemble the sequence reads into contigs. Multilocus sequence typing (MLST), acquired resistance genes, chromosomal mutations, and plasmid incompatibility groups were detected using the CGE database (http://www.genomicepidemiology.org/). PCR analysis and Sanger sequencing were performed to assemble plasmid contigs into a complete plasmid sequence (Supplementary Table S3). Initial analysis and annotation of contigs or plasmids containing *mcr-1* were using the RAST (https://rast.nmpdr.org/rast.cgi), ISfinder (https://www-is.biotoul.fr/), BLAST (https://blast.ncbi.nlm.nih.gov/Blast.cgi) and Gene Construction kit 4.5 (Textco BioSoftware, Inc., Raleigh, NC). The genetic structures of *mcr-1* in plasmids were drawn using Vector NTI 11 (Thermo Fisher Scientific, Inc., Waltham, MA) and manually adjusted. All whole genome sequences and *mcr-1*-carrying plasmids have been deposited in GenBank under the accession numbers PRJNA967092 and PRJNA974499, respectively.

### Conjugation experiments

As mentioned previously, conjugation experiments were performed using all *mcr-1*-positive *E. coli* isolates [14]. The *mcr-1*-positive *E. coli* isolates were used as the donors and high-level streptomycin-resistant *E. coli* C600 was used as the recipient; they were mixed in a ratio of 1:4. Transconjugants were selected on EMB agar containing colistin (2 µg/mL) and streptomycin (3000 µg/mL) and were further confirmed by detecting *mcr-1* using PCR. The experiments were performed in triplicate, and the conjugal frequency of *mcr-1* was estimated as the number of transconjugants per recipient.

## Results

### **Characterization of* mcr-1*-positive* E. coli* isolates**

In total, 1,403 *E. coli* strains were isolated from food-producing animals and retail meat products (Supplementary Table S1). Of these, 13 isolates (0.93%) from chicken meat (*n* = 7), chickens (*n* = 3), and pigs (*n* = 3) exhibited resistance to colistin with minimum inhibitory concentration (MIC) values of 4 to 16 mg/L. No other *mcr* variants were detected, except for *mcr-1* (Table 1). A low detection rate of *mcr-1* among *E. coli* isolates originating from food-producing animals (0.62%, 6/974) and animal-derived food (1.63%, 7/429) was observed in this study.

**Table 1:** Characteristics of *mcr-1*-carrying *E. coli* isolates in this study

Strain^a^	Source	ST	Resistance genes	Colistin MIC (mg/L)	Other resistance patterns^b^	mutations		mcr-1 location	plasmids replicons
						gyrA	parC		
YZ19MCE13	chicken meat, 2019	48	*bla*_CTX−M−123_, *bla*_OXA−1_, *aph(4)-Ia*, Δ*aadA8*, *aph(3’)-IIa*, *aac(3)-IVa*, *catB3*, *aac(6’)Ib-cr*, *fosA*, *mcr-1*, *sul2*, *dfrA12*, *mph(A)*, *arr-3*	4	AMP/CFZ/CTX/GEN/CHL/NAL/CIP/FOS/SXT	S83L D87N	S80I	pYUYZMC13-MCR (IncI2, 59,874 bp)	IncI2, IncFII, IncFIB, IncY, Col156
YZ20MCE6	chicken meat, 2020	6388	*bla*_CTX−M−55_, *aadA2*, *aadA22, strAB*, *tet*(A), *floR*, *fosA*, *mcr-1*, *sul1*, *sul2*, *drfA12*	4	AMP/CFZ/CTX/STR/TET/CHL/FFC/NAL/CIP/FOS/SXT	S83L D87N	S80I	pYUYZMC6-MCR (IncI2, 60,961 bp)	IncI1, IncI2, IncFII, IncFIB, p0111, Col(MG828)
SD20MCE15	chicken meat, 2020	1011	*bla*_TEM−1B_, *bla*_CTX−M−55_, *bla*_OXA−1_, *aadA2*, *aac(3)-IId*, *aph(3’)-Ia*, *strAB*, *tet*(A), *floR*, *catA1*, *catB3*, *oqxAB*, Δ*aac(6’)Ib-cr*, *mcr-1*, *sul1*, *sul2*, *drfA12*, *mph*(A), *arr-3*	8	AMP/CFZ/CTX/GEN/STR/TET/CHL/FFC/NAL/CIP/SXT	S83L D87N	S80I	pYUSDMC15-MCR (IncI2, 63,538 bp)	IncI2, IncFII, IncFIB
YZ19MCE34	chicken meat, 2019	10	*bla*_TEM−1B_, *aac(3)-IId*, *aadA1*, *aadA2*, *aph(3’)-Ia*, *tet*(A), *tet*(M), *cmlA1*, *floR*, *mcr-1*, *sul2*, *sul3*, *dfrA12*, Δ*mef*(B), *lnu*(F)	8	AMP/GEN/STR/TET/CHL/FFC/NAL/CIP/SXT	S83L D87N	S80I	pYUYZMC34-MCR (IncI2, 63,410 bp)	IncI1, IncI2, IncFII, IncFIB
LN19MCE7	chicken meat, 2019	155	*bla*_CTX−M−55_, *bla*_CTX−M−14_, *aadA1*, *aadA2*, *aadA5*, *aac(3)-IVa*, *aph(4)-Ia*, *strAB*, *tet*(A), *cmlA1*, *floR*, *fosA*, *mcr-1*, *sul1*, *sul2*, *drfA17*	16	AMP/CFZ/CTX/GEN/STR/TET/CHL/FFC/NAL/CIP/FOS/SXT	S83L D87N	S80I	pYULNMC7-MCR (IncI2, 63,612 bp)	IncI1, IncI2, IncHI2, IncFII, IncFIB, Col156
YZ19MCE28	chicken meat, 2019	101	*bla*_CTX−M−14_, *bla*_OXA−1_, *aph(3’)-IIa*, *tet(A)*, *catB3*, *aac(6’)Ib-cr*, *arr-3*, *mcr-1*, *sul1*, *mph*(A)	8	AMP/CFZ/CTX/TET/CHL/NAL/CIP	S83L D87Y	S80I	pYUYZMC28-MCR (IncI2, 62,638 bp)	IncFII, IncFIB, IncHI2, IncI2, Col(MG828), Col156
SD20MCE26	chicken meat, 2020	3871	*bla*_CTX−M−55_, *bla*_TEM−1b_, *bla*_CTX−M−14_, *aadA1*, *aadA2*, *aac(3)-IVa*, *aac(3)-IIa*, *aph(4)-Ia*, *aph(3’)-Ia*, *rmtB*, *tet*(A), *floR*, *cmlA1*, *oqxAB*, *qnrS2*, *fosA*, *mcr-1*, *sul1*, *sul2*, *sul3*, *mph*(A)	8	AMP/CFZ/CTX/GEN/STR/AMI/TET/CHL/FFC/NAL/FOS/SXT	None	A56T	contig 49 (IncHI2, 101,306 bp)	IncHI2, IncX1, p0111, Col440I
YZ19PE15	pig, 2019	10	*strAB*, *tet*(A), *mcr-1*	8	STR/TET	None	None	pYUYZP15-MCR (IncX4, 34,541 bp)	IncX1, IncX4, IncFII, IncFIA, IncFIB(K), Col(MG828), ColE10
AH20PE7	pig, 2020	101	*bla*_TEM−1b_, *aadA1*, *aadA2*, Δ*aadA22*, *aac(3)-IId*, *aph(3’)-Ia*, *tet*(M), *tet*(A), *floR*, *cmlA1*, *oqxAB*, *mcr-1*, *sul2*, *sul3*, *dfrA12*, *mph*(A), Δ*mef*(B), *erm(42)*	4	AMP/GEN/STR/TET/CHL/FFC/NAL/CIP/SXT	S83L D87N	S80I	pYUAHP7-MCR (IncX4, 36,021 bp)	IncX1, IncX4, F33:A1:B1, p0111
AH20CE39	chicken, 2020	156	*bla*_CTX−M−55_, *bla*_OXA−10_, *bla*_TEM−1B_, *aadA1*, Δ*aadA22*, *aph(3’)-Ia*, *aph(3’)-IIa*, *aph(4)-Ia*, *aac(3)-IVa*, *strAB*, *tet*(A), *cmlA1*, *floR*, *qnrS1*, *fosA*, *mcr-1*, *sul1*, *sul2*, *dfrA14*, *mph*(A), *arr-3*, *lnu*(F)	4	AMP/CFZ/CTX/GEN/STR/TET/CHL/FFC/NAL/CIP/FOS/SXT	S83L D87N	S80I	pYUAHC39-MCR (IncI2, 63,924 bp)	IncA/C, IncFIB, IncHI2, IncI1, IncI2, IncY, Col(MG828)
AH20CE15	chicken, 2020	1589	*bla*_CTX−M−65_, *bla*_TEM−1B_, *aadA1*, *aadA2*, Δ*aadA22*, *aac(3)-IVa*, *aac(3)-IId*, *aph(4)-Ia*, *aph(3’)-Ia*, *strAB*, *tet*(A), *tet*(M), *floR*, *cmlA1*, *oqxAB*, *mcr-1*, *sul1*, *sul2*, *dfrA12*, *mph*(A), *lnu*(F)	4	AMP/CFZ/CTX/GEN/STR/TET/CHL/FFC/NAL/CIP/SXT	S83L D87N	S80I	contig 17 (IncHI2, 106,757 bp)	IncHI2, IncN, IncFII, IncFIB
AH20PE105	pig, 2020	14,521	*mcr-1*	4	None	None	None	pYUAHP105-MCR (IncI2, 60,733 bp)	IncI2, IncFII, IncFIB
AH20CE37	chicken, 2020	156	*bla*_CTX−M−55_, *bla*_OXA−10_, *bla*_TEM−1B_, *aadA1*, Δ*aadA22*, *aph(3’)-Ia*, *aph(3’)-IIa*, *aph(4)-Ia*, *aac(3)-IVa*, *strAB*, *tet*(A), *floR*, *cmlA1*, *qnrS1*, *fosA3*, *mcr-1*, *sul1*, *sul2*, *dfrA14*, *mph*(A), *arr-3*, lnu(F)	4	AMP/CFZ/CTX/GEN/STR/TET/CHL/FFC/NAL/CIP/FOS/SXT	S83L D87N	S80I	pYUAHC37-MCR (IncI2, 64,657 bp)	IncA/C, IncFIB, IncHI2, IncI1, IncI2, IncY, Col(MG828)

^a^ Different locations are indicated as follows: XJ, Xinjiang Province; YZ, Yangzhou, Jiangsu Province; SD, Shandong Province; LN. Liaoning Province; AH, Anhui Province;

^b^ AMP, ampicillin; CFZ, cefazolin; CTX, cefotaxime; GEN, gentamicin; AMI, amikacin; TET, tetracycline; CHL, chloramphenicol; FFC, florfenicol; NAL, nalidixic acid; CIP, ciprofloxacin; FOS, fosfomycin; SXT, sulfamethoxazole/trimethoprim; STR, streptomycin

As shown in Table 1, all colistin-resistant *E. coli* isolates, except for isolate AH20PE105, were also resistant to multiple antibiotics. Eleven isolates were resistant to ampicillin, tetracycline, and chloramphenicol. Additionally, among the 13 *mcr-1*-positive isolates, resistance to streptomycin, sulfamethoxazole/trimethoprim, and gentamicin was observed in 10, 10, and nine cases, respectively. Nine isolates contained *bla*_CTX−M_, including *bla*_CTX−M−55_ (*n* = 6), *bla*_CTX−M−14_ (*n* = 3), *bla*_CTX−M−65_ (*n* = 1), and *bla*_CTX−M−123_ (*n* = 1), and were resistant to cefazolin and cefotaxime. Two isolates, SD20MCE26 and LN19MCE7, carried both *bla*_CTX−M−55_ and *bla*_CTX−M−14_. Furthermore, nine MCR-1-producing *E. coli* strains exhibited resistance to florfenicol, a common veterinary antimicrobial agent, and carried the florfenicol resistance gene *floR*. The fosfomycin resistance gene *fosA3* and 16S rRNA methylase gene *rmtB* were identified in six fosfomycin-resistant strains and one amikacin-resistant strain, respectively. In addition, *gyrA* (S83L and D87N/Y) and *parC* (S80I) mutations were observed in 10 *E. coli* strains, thereby explaining their nalidixic acid- and ciprofloxacin-resistant phenotypes. The presence of the multidrug efflux pump genes *oqxAB* and quinolone resistance gene *qnrS2* and the existence of a single mutation in *parC* (A56T) in isolate SD20MCE26 were responsible for its resistance to nalidixic acid.

MLST analysis based on whole genome sequencing revealed that 13 *mcr-1*-positive *E. coli* isolates belonged to 10 sequence types (STs), including ST10 (*n* = 2), ST101 (*n* = 2), ST156 (*n* = 2), ST155, ST48, ST6388, ST1011, ST3871, ST1589, and a new ST (ST14521) (Table 1). Three to seven plasmid replicons were identified in *mcr-1*-positive *E. coli* strains (Table 1). By analyzing *mcr-1*-carrying contigs or plasmids, we found that *mcr-1* was located on plasmids in all isolates and that IncI2 was predominant (*n* = 9), followed by IncX4 (*n* = 2) and IncHI2 (*n* = 2) (Table 1).

### **Characterization of* mcr-1*-carrying IncI2 plasmids in nine* E. coli* isolates**

**Fig. 1:**
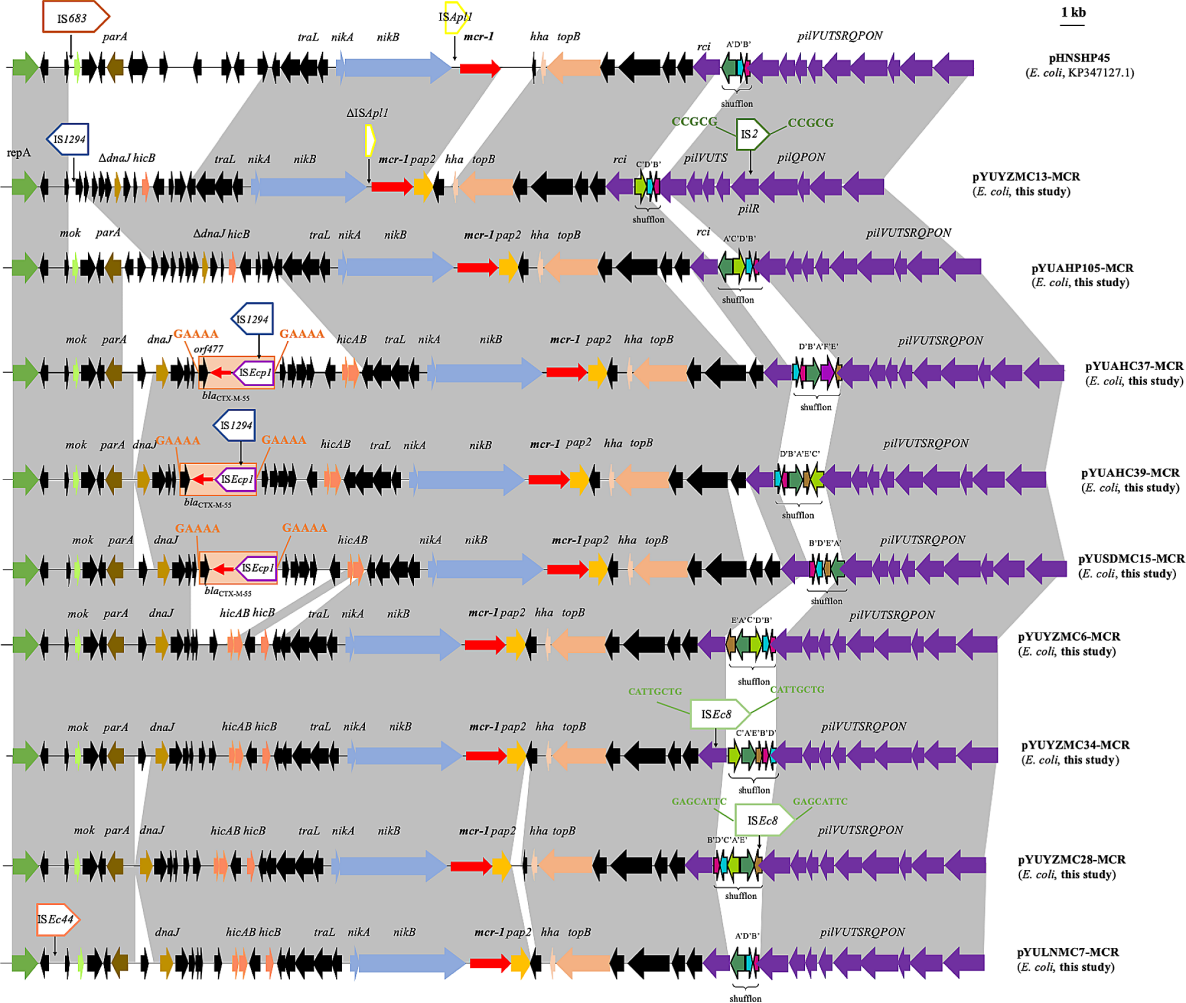
Genetic structure of *mcr-1* in nine plasmids and structural comparison with related *mcr-1*-carrying IncI2 plasmids. Regions with > 99% identity are shaded in gray. Thick arrow indicates ORF. Red thin red arrow indicates resistance genes. Box indicates insertion sequences. The delta symbol (Δ) indicates a truncated gene or mobile element. Direct repeats are indicated by arrows and sequences

The *mcr-1*-carrying IncI2 plasmids in this study were similar to the first reported *mcr-1*-carrying plasmid pHNSHP45 (*E. coli*, pig, China, KP347127) (Fig. 1). The transposable element IS*Apl1* observed upstream of *mcr-1* in pHNSHP45 was not present in eight *mcr-1*-carrying plasmids. Moreover, only one plasmid (pYUYZMC13-MCR) from the isolate YZ19MCE13 contained an incomplete IS*Apl1* upstream of *mcr-1* (Fig. 1). In addition to *mcr-1*, the plasmids pYUAHC37-MCR, pYUAHC39-MCR, and pYUSDMC15-MCR from the isolates AH20CE37, AH20CE39, and SD20MCE15, respectively, carried *bla*_CTX−M−55_. It was located in a typical transposition unit (IS*Ecp1*–*bla*_CTX−M−55_–*orf477*) with 5-bp direct repeats (DRs, 5′-GAAAA-3′), while IS*Ecp1* was interrupted by IS*1294* in pYUAHC37-MCR and pYUAHC39-MCR (Fig. 1).

A multiple inversion system called shufflon was first identified in the IncI1 plasmid R64 and later discovered in other Inc types, including IncIγ, IncI2, IncK, and IncZ plasmids [15]. The rearrangement of shufflon regions results in the generation of different C-terminal ends of the PilV protein, which is involved in bacterial conjugation [15]. The shufflon region of nine IncI2 plasmids was distinct and included eight arrangements, and it was interrupted by IS*Ec8* in pYUYZMC28-MCR (Fig. 1). A similar IS*Ec8* insertion was observed in the recombinase gene *rci* in pYUYZMC34-MCR (Fig. 1). In pYUYZMC13-MCR, the *mok*–*parA* fragment (2007 bp) was absent because of the insertion of IS*1294* and the type IV pilus transmembrane gene *pilR* was interrupted by IS*2*, generating 5-bp DRs (5′-CCGCG-3′) (Fig. 1). The insertion of mobile elements into the conjugal region may explain the failure of conjugation of the three abovementioned *mcr-1*-carrying plasmids. Three plasmids in this study (pYUYZMC6-MCR, pYUSDMC15-MCR, and pYUAHP105-MCR) could be successfully transferred to *E. coli* C600 at a frequency of 5.79 × 10^− 6^, 8.99 × 10^− 3^, and 1.9 × 10^− 4^ transconjugants per recipient, respectively.

### **Characterization of* mcr*-1-carrying IncX4 plasmids in two* E. coli* isolates**

**Fig. 2:**
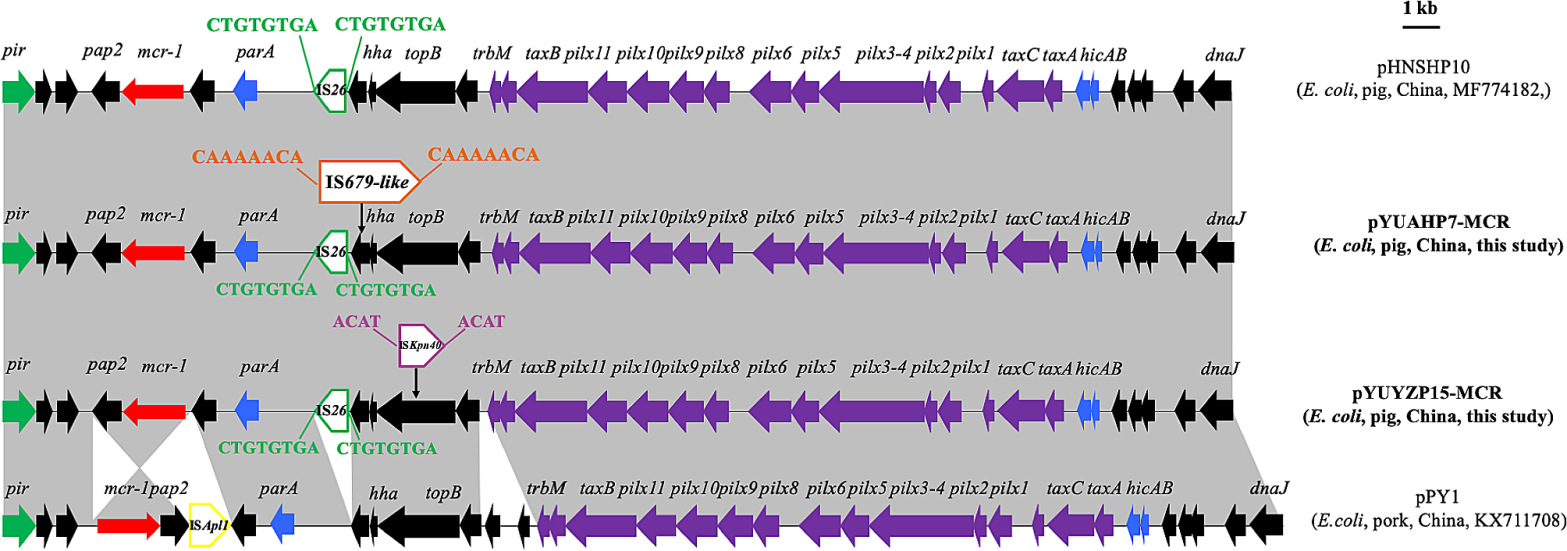
Genetic structure of *mcr-1* in two plasmids and structural comparison with related *mcr-1*-carrying IncX4 plasmids. Regions with > 99% identity are shaded in gray. Red arrow indicates resistance genes. Purple arrow represents conjugal transfer genes. Green arrow represents plasmid replication genes. Blue arrow represents genes for maintenance and stability. Black arrow indicates other genes. The delta symbol (Δ) indicates a truncated gene or mobile element. Direct repeats are indicated by arrows and sequences

Two *mcr-1*-carrying IncX4 plasmids, pYUAHP7-MCR and pYUYZP15-MCR, were obtained from isolates AH20PE7 and YZ19PE15, respectively, with a size of 34,541–36,021 bp. Both plasmids had typical IncX4 plasmid backbones, including genes encoding replication proteins and conjugal transfer proteins and those responsible for maintenance and stability (Fig. 2). Moreover, their organization was similar to that of other *mcr-1*-carrying IncX4 plasmids from animals or food products in China, such as pHNSHP10 (pig, MF774182) and pPY1 (pork, KX711708) from *E. coli* (Fig. 2). However, IS*Apl1* was inserted downstream of *mcr-1*–*pap2* in pY1, which was not present in our plasmids. Moreover, the *mcr-1*–*pap2* segment was inserted in pPY1 in reverse orientation. Instead, one copy of IS*26* was inserted into the backbone of the two plasmids at the same site, generating 8-bp DRs (5′-CTGTGTGA-3′) (Fig. 2). In addition to IS*26*, one copy of IS*679-like* was inserted into *hns* with 8-bp DRs in pYUAHP7-MCR. Moreover, *topB* was interrupted by the insertion of IS*Kpn40* in pYUYZP15-MCR (Fig. 2). As described previously [4, 16, 17], the IncX4 plasmids pYUAHP7-MCR and pYUYZP15-MCR did not carry any drug resistance genes, except for *mcr-1*. They were transferable at a frequency of 3.75 × 10^− 4^ and 2.03 × 10^− 3^ transconjugants per recipient, respectively.

### **Characterization of* mcr-1*-carrying contigs associated with IncHI2 plasmids in two* E. coli* isolates**

**Fig. 3:**
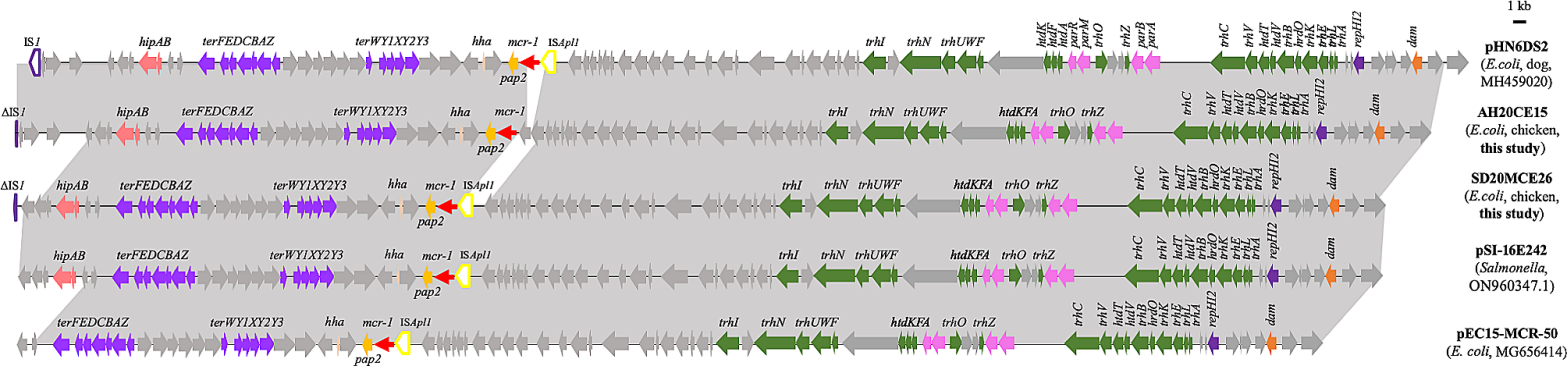
Genetic structure of *mcr-1* in SD20MCE26 and AH20CE15 in this study and comparison with other IncHI2 plasmids. Regions with > 99% identity are shaded in gray. Thick arrow indicates ORF. Red thin red arrow indicates resistance genes. Box indicates insertion sequences

In strains SD20MCE26 and AH20CE15, *mcr-1*-carrying contigs (101,306 bp and 106,757 bp, respectively) were highly similar to the IncHI2 plasmids pHN6DS2 (*E. coli*, MH459020), pSI-16E242 (*Salmonella*, ON960347), and pEC15-MCR-50 (*E. coli*, MG656414) (Fig. 3). The *mcr-1*-positive contigs in SD20MCE26 and AH20CE15 harbored tellurium resistance genes (*terYXWZABCDEF*) and genes responsible for conjugal transfer (*trhINUWFOZCVBKELA* and *htdKFATVO*). The insert sequence IS*Apl1* observed upstream of *mcr-1* in pHN6DS2 was present in SD20MCE26 but absent in AH20CE15 (Fig. 3). As IncHI2 plasmid replicons were identified in SD20MCE26 and AH20CE15 (Table 1) and *mcr-1* from SD20MCE26 and AH20CE15 could be transferred to *E. coli* C600 at a frequency of 3.24 × 10^− 5^ and 1.94 × 10^− 5^ transconjugants per recipient, respectively, *mcr-1* in SD20MCE26 and AH20CE15 may be associated with IncHI2 plasmids.

## Discussion

Since its identification in 2015 in a porcine *E. coli* strain in China, the plasmid-mediated colistin resistance gene *mcr-1* has rapidly disseminated worldwide, being identified in diverse bacterial species across various sources, predominantly hosted by *E. coli* [1–4, 8]. In our study, a low prevalence of *mcr-1* was observed among *E. coli* isolates from food-producing animals and animal-derived food. It might be attributed to the random isolation of *E. coli* strains without employing a medium supplemented with colistin for selection. Furthermore, we identified colistin-resistant *E. coli* isolates from chickens (1.29%, 3/275) and pigs (1.09%, 3/232), while none were found in cattle (0/507) or pigeons (0/27). The absence in pigeons might be due to the limited numbers of *E. coli* isolates, and notably, colistin has never been approved for use in cattle in China. On the other hand, our findings align with the substantial decrease in colistin resistance and *mcr* prevalence following the ban of colistin-positive additives in China [9]. This underscores the critical significance and consequential effects of prohibiting colistin as an animal growth promoter in China. Although a low detection rate of *E. coli* isolates carrying *mcr-1* gene was noted in this study, its presence in animals and food remains a considerable threat to public health, given the potential risk of zoonotic transmission to humans through the food chain and contact with backyard animals [1, 7]. In addition to *mcr-1*, 12 isolates investigated in this study contained multiple antimicrobial resistance genes, such as *bla*_CTX−M_, *floR*, *oqxAB*, *qnrS*, and mutations in *gyrA* and *parC*, consistent with their antibiotic-resistant phenotype. The emergence and spread of these MDR *E. coli* strains present a heightened risk, potentially resulting in difficult-to-treat infections and limiting therapeutic options against infections they cause [7]. More importantly, they serve as a significant reservoir of resistance determinants to most families of antimicrobial agents for animals and humans [7].

A high diversity of *E. coli* isolates with different STs carrying *mcr-1* has been identified in animals, food products, and humans [4, 9, 16]. The STs identified in this study, such as ST10, ST48, ST101, ST155, ST156, ST1011 and ST1589, were also previously reported as common *mcr-1* carriers in food-producing animals and humans in China [9, 16, 18]. These STs have also been recognized as *mcr-1* carriers beyond China. For example, *mcr-1*-positive *E. coli* ST10, ST1011, and ST156 were identified in poultry samples from Poland [19]. In Egypt, two *E. coli* ST155 strains encoding Tet(X7) and MCR-1 were isolated from chicken meat, while three *mcr-1-*positive *E. coli* ST101 strains were recovered from pigs in Europe [20, 21]. The diversity observed in *E. coli* STs in our study and prior research suggests that horizontal transmission serves as the primary route for *mcr-1* dissemination in animals and their food products. Nonetheless, it is noteworthy that the clonal spread of specific ST-type *E. coli* strains, such as *E. coli* ST93 among companion animals, and *E. coli* ST10 in swine farms, may also contribute to the spread of *mcr-1* [22, 23].

Plasmids play an essential role in the global dissemination of resistance genes including *mcr-1* in *Enterobacteriaceae* [1, 3, 4, 24]. While various plasmids, such as IncFII, IncY, IncP, and IncK2, have been described as vectors of *mcr-1*, the majority of identified plasmids were affiliated with three incompatibility groups: IncI2, IncX4, and IncHI2 [3, 4, 8, 17, 25]. These three prevalent plasmid types have served as the principal vehicles for disseminating *mcr-1* globally, frequently detected in *Enterobacteriaceae*, particularly *E. coli*, across diverse origins [3, 4, 8]. Consistent with this trend, our study also detected the presence of *mcr-1* on IncI2, IncX4, or IncHI2 plasmids, with high similarity to each other and previously reported *mcr-1*-carrying plasmids within the same incompatibility group. Furthermore, a diverse array of IS elements was found integrated into the plasmid backbone, leading to the loss or acquisition of genetic fragments and driving plasmid evolution among different lineages of *E. coli* strains. Seven *mcr-1*-carrying plasmids identified in our study possess conjugative capabilities, representing an increased risk of spreading *mcr-1* between bacteria, even across different species. This conjugal transferability significantly contributes to the dissemination of both *mcr-1* and colistin resistance among bacterial populations [8].

In this study, IncI2 plasmids emerged as the primary vehicle for *mcr-1* transmission, sharing a similar backbone yet distinct shufflon regions. Intriguingly, a striking rise in the occurrence of IncI2-type plasmids was noticed among *mcr-1*-positive *E. coli* strains from animals and humans following the cessation of colistin as an animal growth promoter in China [9]. The precise reason behind this observation remains unclear. However, IncI2 plasmids often carry additional resistance genes such as *bla*_CTX−M−55_ in this study, besides *mcr-1*, potentially contributing to the preferential selection of these plasmids due to the extensive use of β-lactam antibiotics such as amoxicillin in animals and cephalosprins in human clinical settings [9]. Furthermore, the enhanced fitness conferred by *mcr-1*-carrying IncI2 and IncX4 plasmids supports their dissemination and persistence in bacterial populations even without antibiotic selection pressures [17]. Conversely, the acquisition of *mcr-1*-carrying IncHI2 plasmids imposes a competitive disadvantage [17, 26]. Nonetheless, these plasmids often carry diverse resistance genes (e.g., *floR*, *bla*_CTX−M_, and *fosA3*), co-selection by other antimicrobials might augment the further dissemination of IncHI2 plasmids carrying *mcr-1* [8, 17, 22, 27].

The insertion sequence IS*Apl1* is involved in mobilizing *mcr-1* among DNA molecules, such as plasmid or chromosomes [3, 4, 25, 28]. However, in this study, the presence of IS*Apl1*, intact or incomplete, was observed upstream of *mcr-1* in only two plasmids, while the *mcr-1*-*pap2* structure (*n* = 11) was more prevalent. This observation aligns with a prior study indicating that IS*Apl1* upstream of the *mcr-1* gene was present in 77.8% of IncHI2 plasmids, 37.9% of IncI2 plasmids, and absent in IncX4 plasmids [4]. The proposed hypothesis suggests that *mcr-1* initially mobilizes through the mobile transposon unit Tn*6330* (IS*Apl1*-*mcr-1*-*pap2*-IS*Apl1*), subsequently undergoing a gradual loss of IS*Apl1* at both ends, potentially ensuring the stability of *mcr-1* before plasmid-mediated transmission [3, 29]. Consequently, this results in the formation of diverse genetic structures harboring *mcr-1*, with the *mcr-1-pap2* structure being predominant, followed by the IS*Apl1*-*mcr-1*-*pap2* structure [3, 29].

However, our study has several limitations. We collected samples from only seven regions in China, however, certain sources (e.g., pigeons and beef) were limited in sample quantity and location. The uneven distribution of samples across different regions and types led to considerable variation in the number of strains isolated from different areas. For instance, there were notably fewer pig and pork samples and isolated strains from Guangdong province. Additionally, we exclusively detected *mcr* genes in colistin-resistant *E. coli* isolates. Notably, *mcr-4.3* demonstrated a silent phenotype due to mutations (V179G and V236F), and silent transmission of inactivated *mcr-1* and *mcr-9* with inducible colistin resistance have been previously reported [30–33]. Hence, there remains a possibility that the colistin-susceptible *E. coli* strains yet to be identified in our study may carry *mcr*.

## Conclusion

This study unveils a low detection rate (0.93%) of *mcr-1* among *E. coli* isolates originating from food-producing animals and animal-derived food products, associated with the previously identified IncI2, IncX4 and IncHI2 epidemic plasmids. While the low prevalence of *mcr-1* might not immediately appear threatening, its emergence merits attention given its implication for colistin resistance and public health. Considering the potential dissemination of *mcr-1* facilitated by plasmids among bacteria, and the risk of co-selection with other commonly used antibiotics in animal husbandry, continuous surveillance of *mcr-1* is imperative. This surveillance needs to monitor not only its prevalence and dissemination in *E. coli* but also in other *Enterobacteriaceae* species.

### Electronic supplementary material

Below is the link to the electronic supplementary material.

Supplementary Material 1

## Data Availability

The datasets used and/or analyzed during the current study available from the corresponding author on reasonable request. All whole genome sequences and *mcr-1*-carrying plasmids are available in GenBank under the accession numbers PRJNA967092 and PRJNA974499, respectively.
