# Supplementary material for: Low prevalence of mcr-1 in Escherichia coli from food-producing animals and food products in China
Source: BMC Vet Res. 2024 Feb 1;20:40. doi: 10.1186/s12917-024-03891-6 (PMC10832210; doi:10.1186/s12917-024-03891-6)
Supplement: Supplementary file 1 — Supplementary Material 1 [file 12917_2024_3891_MOESM1_ESM.docx]

**Table S1 *E. coli* isolates obtained in this study**

| **Source** | **Location** | **Sampling Time** | **Number of samples** | **Number of *E. coli* isolates** |
| --- | --- | --- | --- | --- |
| cattle | Jiangsu | 2019.06 | 121 | 94 |
|  | Jiangsu | 2019.07 | 159 | 137 |
|  | Liaoning | 2019.07 | 224 | 121 |
|  | Xinjiang | 2019.08 | 115 | 65 |
|  | Xinjiang | 2020.11 | 133 | 90 |
| pig | Liaoning | 2019.07 | 69 | 45 |
|  | Guangdong | 2019.09 | 8 | 8 |
|  | Anhui | 2020.11 | 135 | 112 |
| chicken | Shandong | 2019.08 | 42 | 22 |
|  | Xinjiang | 2019.08 | 97 | 79 |
|  | Henan | 2020.05 | 70 | 58 |
|  | Anhui | 2020.11 | 149 | 116 |
| pigeon | Xinjiang | 2019.08 | 31 | 27 |
| beef | Jiangsu | 2019.07 | 39 | 14 |
|  | Liaoning | 2019.07 | 68 | 20 |
|  | Shanghai | 2019.08 | 11 | 8 |
| pork | Jiangsu | 2019.07 | 164 | 138 |
|  | Liaoning | 2019.07 | 43 | 13 |
|  | Shanghai | 2019.08 | 59 | 45 |
|  | Shandong | 2019.08 | 18 | 11 |
|  | Guangdong | 2019.09 | 1 | 1 |
|  | Jiangsu | 2020.05 | 18 | 2 |
|  | Shandong | 2020.06 | 46 | 20 |
|  | Anhui | 2020.11 | 28 | 13 |
| chicken meat | Jiangsu | 2019.07 | 60 | 33 |
|  | Liaoning | 2019.07 | 66 | 10 |
|  | Shanghai | 2019.08 | 24 | 18 |
|  | Shandong | 2019.08 | 17 | 7 |
|  | Xinjiang | 2019.08 | 16 | 5 |
|  | Jiangsu | 2020.05 | 38 | 15 |
|  | Shandong | 2020.06 | 85 | 52 |
|  | Anhui | 2020.11 | 35 | 4 |
| **Total** |  |  | **2189** | **1403** |

**Table S2 Primers used for PCR in this study**

| **Gene** | **Primer name** | **Sequence(5’ to 3’)** | **Size (bp)** | **Reference** |
| --- | --- | --- | --- | --- |
| *mcr-1* | mcr-1-F | TGCCAATCTACTCGG | 536 | This study |
|  | mcr-1-R | GTCATCTAAGCCAACG |  |  |
| *mcr-2* | mcr-2-F | TGGTACAGCCCCTTTATT | 1617 | [1] |
|  | mcr-2-R | GCTTGAGATTGGGTTATGA |  |  |
| *mcr-3* | mcr-3-F | TTGGCACTGTATTTTGCATTT | 542 | [2] |
|  | mcr-3-R | TTAACGAAATTGGCTGGAACA |  |  |
| *mcr-4* | mcr-4-F | ATTGGGATAGTCGCCTTTTT | 487 | [3] |
|  | mcr-4-R | TTACAGCCAGAATCATTATCA |  |  |
| *mcr-5* | mcr-5-F | ATGCGGTTGTCTGCATTTATC | 1644 | [4] |
|  | mcr-5-R | TCATTGTGGTTGTCCTTTTCTG |  |  |
| *mcr-6* | mcr-6-F | AGCTATGTCAATCCCGTGAT | 252 | [5] |
|  | mcr-6-R | ATCACGGGATTGACATAGCTAC |  |  |
| *mcr-7* | mcr-7-F | GCCCTTCTTTTCGTTGTT | 551 | [5] |
|  | mcr-7-R | GGTTGGTCTCTTTCTCGT |  |  |
| *mcr-8* | mcr-8-F | TCAACAATTCTACAAAGCGTG | 856 | [5] |
|  | mcr-8-R | AATGCTGCGCGAATGAAG |  |  |
| *mcr-9* | mcr-9-F | TTCCCTTTGTTCTGGTTG | 1011 | [5] |
|  | mcr-9-R | GCACCTAATAAGTCGGTC |  |  |
| *mcr-10* | mcr-10-F | GGACCGACCTATTACCAGCG | 366 | [6] |
|  | mcr-10-R | GGCATTATGCTGCAGACACG |  |  |

**References**

[1] Xavier BB, Lammens C, Ruhal R, Kumar-Singh S, Butaye P, Goossens H, et al. Identification of a novel plasmid-mediated colistin-resistance gene, *mcr-2*, in *Escherichia coli*, Belgium, June 2016. Euro Surveill 2016;21(27).

[2] Yin W, Li H, Shen Y, Liu Z, Wang S, Shen Z, et al. Novel plasmid-mediated colistin resistance gene *mcr-3* in *Escherichia coli*. mBio 2017;8(3):e00543-17.

[3] Carattoli A, Villa L, Feudi C, Curcio L, Orsini S, Luppi A, et al. Novel plasmid-mediated colistin resistance mcr-4 gene in Salmonella and Escherichia coli, Italy 2013, Spain and Belgium, 2015 to 2016. Euro Surveill 2017;22(31):30589.

[4] Borowiak M, Fischer J, Hammerl JA, Hendriksen RS, Szabo I, Malorny B. Identification of a novel transposon-associated phosphoethanolamine transferase gene, *mcr-5*, conferring colistin resistance in d-tartrate fermenting *Salmonella* enterica subsp. enterica serovar Paratyphi B. J Antimicrob Chemother 2017;72(12):3317-3324.

[5] Borowiak M, Baumann B, Fischer J, Thomas K, Deneke C, Hammerl JA, et al. Development of a novel *mcr-6* to *mcr-9* multiplex PCR and assessment of *mcr-1* to *mcr-9* occurrence in colistin-resistant *Salmonella* enterica isolates from environment, feed, animals and food (2011-2018) in Germany. Front Microbiol 2020;11:80.

[6] Lei CW, Zhang Y, Wang YT, Wang HN. Detection of mobile colistin resistance gene *mcr-10.1* in a conjugative plasmid from *Enterobacter roggenkampii* of chicken origin in China. Antimicrob Agents Chemother 2020;64(10):e01191-20.

**Table S3** **Primers used to assemble *mcr-1*-carrying plasmids**

| **Region** | **sequence (5'-3')** | **Size (bp)** | **Position (bp) in plasmid** |
| --- | --- | --- | --- |
| IS*26*-*hns* | F: GTCAGAGCCACCAAC | 1406 (pYUAHP7-MCR)  1421(pYUYZP15-MCR) | 8777-8791 (pYUAHP7-MCR)  8777-8791 (pYUYZP15-MCR) |
|  | R: AAAGGCGGAGTAAGA |  | 10168-10182 (pYUAHP7-MCR)  10183-10197 (pYUYZP15-MCR) |
| *∆hns*-IS*679*-like-*∆hns-hha* | F: CGGGATCAAGACCTAA | 2998 | 10236-10251 (pYUAHP7-MCR) |
|  | R: CGGCGTCTGGAAGTA |  | 13219-13233 (pYUAHP7-MCR) |
| *∆topB*-IS*Kpn40*-*∆topB* | F: TCTCCTGTGCGAATT | 1603 | 11463-11477 (pYUYZP15-MCR) |
|  | R: ATAACCGCTCTGACTG |  | 13109-13124 (pYUYZP15-MCR) |
| *∆pilR*-IS*2*-*∆pilR* | F: TATGGTATCGTGGACC | 1635 | 32138-32153 (pYUYZMC13-MCR) |
|  | R: CAGACTACGGGAAGAA |  | 33757- 33772 (pYUYZMC13-MCR) |
| *rci*-*pilV* | F: GCGTTCAGCAATAGG | 2161 (pYUAHP105-MCR)  2428 (pYUYZMC6-MCR)  1927 (pYUSDMC15-MCR)  2477 (pYUAHC37-MCR) | 29014-29028 (pYUAHP105-MCR)  29602-29616 (pYUYZMC6-MCR)  32224-32238 (pYUSDMC15-MCR)  32925-32939 (pYUAHC37-MCR) |
|  | R: CAGGAAATACAGCCAC |  | 31159-31174 (pYUAHP105-MCR)  32014-32029 (pYUYZMC6-MCR)  34135-34150 (pYUSDMC15-MCR)  35386-35401 (pYUAHC37-MCR) |
| *∆pilV*-IS*Ec8*-*∆pilV* | F:ACCTAATGCCCTCCA | 2923 | 30655- 30669 (pYUYZMC28-MCR) |
|  | R:GCAGCGTGAATAATAAA |  | 33561- 33577 (pYUYZMC28-MCR) |
| *∆*IS*Ecp1*-IS*1294*-*∆*IS*Ecp1* | F: ATTGTAGCATCGGTTTC | 2001 | 10267-10283 (pYUAHC37-MCR)  9534-9550 (pYUAHC39-MCR) |
|  | R: GCTCTGCGGTCACTT |  | 12253-12267 (pYUAHC37-MCR)  11520-11534 (pYUAHC39-MCR) |
